# Participation of the adrenal gland in the anti-inflammatory effect of polyunsaturated diets

**DOI:** 10.1155/S0962935195000585

**Published:** 1995-09

**Authors:** V. L. F. Silveira, E. A. Limãos, D. W. Nunes

**Affiliations:** 1 Department of Physiology Escola Paulista de Medicina Universidade Federal de São Paulo São Paulo SP Brazil

## Abstract

The effect of an n-3 (fish) and n-6 (soybean) fatty acid-rich diet on carrageenin paw oedema in rats, and the participation of adrenal gland, corticosterone and α_2_-macroglobulin (α_2_-M) in this process were studied. A significant inhibition of carrageenin oedema was observed not only in rats fed a diet rich in fish oil but also in the soybean group. α_2_-M was not detectable before carrageenin injection, suggesting that this putative antiinflammatory factor does not participate in the observed anti-inflammatory effect. Corticosterone levels were higher in fat-fed than in control rats, before carrageenin stimulus and adrenalectomy abolished the anti-inflammatory response in fat-fed animals, showing the important role of the adrenocortical hormones in this process.


Mediators of Inflammation 4, 359–363 (1995)

### Participation of the adrenal gland in the anti-inflammatory effect of polyunsaturated diets

V. L. F. Silveira,[CA] E. A. Limãos and D. W. Nunes

Department of Physiology, Escola Paulista de Medicina, Universidade Federal de São Paulo, São Paulo, SP, Brasil

[CA]Corresponding Author

**THE effect of an n-3 (fish) and n-6 (soybean) fatty acid-rich diet on carrageenin paw oedema in rats, and the participation of adrenal gland, corticosterone and $\alpha_2$-macroglobulin ($\alpha_2$-M) in this process were studied. A significant inhibition of carrageenin oedema was observed not only in rats fed a diet rich in fish oil but also in the soybean group. $\alpha_2$-M was not detectable before carrageenin injection, suggesting that this putative anti-inflammatory factor does not participate in the observed anti-inflammatory effect. Corticosterone levels were higher in fat-fed than in control rats, before carrageenin stimulus and adrenalectomy abolished the anti-inflammatory response in fat-fed animals, showing the important role of the adrenocortical hormones in this process.**

**Key words:** Carrageenin oedema, Corticosteroids, $\alpha_2$-Macroglobulin, Polyunsaturated fatty acids.

### Introduction

The fatty acids of membrane phospholipids act as precursors of several biologically active mediators. Arachidonic acid, an n-6 polyunsaturated fatty acid (n-6 PFA), is a substrate for both cyclooxygenase, which leads to the pro-inflammatory prostaglandins (Pg) and thromboxanes (Tx); and lipoxygenase, which causes the production of leukotrienes (Lt), which are important mediators of the inflammatory response.[1,2] It is now well established that the fatty acid composition of membrane phospholipids can be modified by some dietary components. As n-3 polyunsaturated fatty acids (n-3 PFA) are rapidly and preferentially incorporated into membrane phospholipids, the presence of large quantities of these fatty acids in the diet results in reduced production of arachidonic acid metabolites. In this situation products with less potent inflammatory activity are produced.[1,3,4]

It has been suggested that this lower level of arachidonic acid-derived mediators exerts a beneficial effect on some inflammatory processes.[5] Indeed, several studies have shown that the severity of rheumatoid arthritis is reduced when high quantities of n-3 PFA are given.[6–8] Fish oil that is rich in n-3 PFA has been associated with reduction in mortality from coronary artery disease.[9] On the other hand, evidence that fish oil administration enhances collagen-induced arthritis in rats and exacerbates auto-immune vasculitis in mice[10] have dictated caution in premature use of fish oil treatments in inflammatory diseases.

Changes in the lipid composition of cell membranes may also alter the production of non-lipid inflammatory mediators such as interleukin-1 (IL-1) and tumour necrosis factor (TNF).[11] IL-1 has been shown to stimulate the secretion of glucocorticoids from the adrenal gland, an effect mediated by locally released catecholamines.[12,13] On the other hand, glucocorticoids have been shown to alter the production of cytokines, such as TNF-$\alpha$ and IL-1.[14] An inhibitory effect of glucocorticoids on the *in vitro* release of TNF[15] and cytokines[16] has been demonstrated. Glucocorticoids are known to induce synthesis of lipocortins,[17] which in turn have been demonstrated to inhibit phospholipase $A_2$[18] with decreased arachidonic acid and Pg production.

In a previous paper, we showed that the presence of adequate levels of adrenal hormones are essential for the production of acute phase reactants.[19] In another study, utilizing the rat paw oedema model we also demonstrated that $\alpha_2$-macroglobulin ($\alpha_2$-M) is an important anti-inflammatory factor acting on the counter-irritation phenomenon and that this effect is mediated by glucocorticoids.[20]

Although the inflammatory process has been associated with glucocorticoids, acute phase reactants, and arachidonic acid metabolites, the participation of these factors on the inflammatory response of animals fed different lipid diets has not been determined. Consequently, the present study was aimed at investigating the effects of an n-6 or n-3 PFA-rich diet on acute inflammatory oedema, and the participation of adrenal gland, corticosterone and $\alpha_2$-M on this process.

© 1995 Rapid Science Publishers

### Materials and Methods

*Animals and treatment:* Male Wistar rats (28–34 days old) weighing approx. 70 g were used. They were housed under controlled condition of light (light on from 7:00 am to 7:00 pm) with free access to chow and water. Body weight and food intake were determined weekly. For 48–53 days the rats received one of three types of diet: (1) standard balanced chow with 4% fat and 22% protein (Nuvilab) (control group); (2) n-6 polyunsaturated fat-rich diet prepared by adding 15% of soybean oil to control chow (soybean group); and (3) n-3 polyunsaturated fat-rich diet prepared by adding 15% of fish oil (Sigma) to control chow (fish group). Soybean and fish oil are rich in fatty acids of the n-6 and n-3 type, respectively. Owing to the addition of fat, 12% of casein (Sigma) was also added to diets 2 and 3, to prevent these diets being hypoproteic relative to control chow.

*Measurement of plasma corticosterone and $\alpha_2$-M:* Blood samples were collected from the tail vein, 1 week before the oedema experiment. Trisodium citrate at a final concentration of 23 mM was used as anticoagulant. Corticosterone was estimated by a fluorometric method.[21]

$\alpha_2$-M was determined by radial immunodiffusion.[22] Because pure $\alpha_2$-M as standard was not available, the results were compared to plasma from turpentine injured rats to which a value of 100 arbitrary units (a.u.) of $\alpha_2$-M was ascribed.[20]

*Bilateral adrenalectomy:* The adrenal glands were removed under pentobarbital sodium anaesthesia (50 mg/ml i.p.) by the dorsal approach.[23] The adrenalectomized rats were supplied with saline *ad libitum* and were used 6–7 days after surgery. To check the success of the adrenalectomy, plasma corticosterone was determined.

*Production and measurement of paw oedema:* Carrageenin (Sigma type IV) was dissolved in distilled water, (1.0 mg/ml) and 0.1 ml of this solution was injected into the sub-plantar area of the hind paw. All animals were maintained under light anaesthesia (sodium pentobarbital, 40 mg/kg, i.p.), throughout the experiments. Paw volumes were determined hourly by phlethysmography (H. Basile, Milan, Italy). Each measurement was repeated three times and the mean value calculated. The period of observation was 4 h.

*Statistical analysis:* Data were expressed as mean values ± S.E.M. Statistical analysis was performed using ANOVA followed by the Duncan's test for multiple comparison. Significance was set at the $p < 0.05$ level.

### Results

*Food intake and body weight:* The food intake results were expressed as g/rat/day and the body weight as percentage increase of initial weight. The food intake was significantly reduced in both soybean and fish groups when compared to the control group. The soybean group had its food intake reduced after the third week and the fish group had a reduction after the first week of treatment (Table 1). A significant increase of body weight was observed in soybean rats after the fourth week and in fish group after the first week (Table 2).

*Carrageenin oedema, $\alpha_2$-M, and corticosterone levels:* Results of carrageenin oedema were expressed as the percentage increase from basal value, to correct for variability between initial paw volume. A significant inhibition of the carrageenin oedema was observed in the soybean and fish groups as compared with control rats. The carrageenin oedema of the soybean group was even smaller than that of the fish group (Fig. 1 and Table 3).

$\alpha_2$-M was not detectable before the oedema experiment in soybean, fish or control rats

Table 1. Food intake (g/rat/day) of rats fed the three diets, during the first 6 weeks

| Diet | Week | | | | | |
|---|---|---|---|---|---|---|
| | 1 | 2 | 3 | 4 | 5 | 6 |
| Control | 13.17 ± 0.77 (13) | 16.49 ± 1.07 (15) | 21.17 ± 0.95 (14) | 22.03 ± 0.67 (16) | 23.17 ± 0.68 (14) | 23.94 ± 0.70 (16) |
| Soybean | 11.46 ± 0.51 (14) | 14.17 ± 0.76 (17) | 14.91* ± 0.39 (16) | 14.78* ± 0.55 (18) | 16.27* ± 0.53 (16) | 16.77* ± 0.80 (18) |
| Fish | 8.20* ± 0.57 (13) | 11.60* ± 0.34 (13) | 14.67* ± 0.38 (13) | 17.36* ± 0.45 (13) | 17.63* ± 0.53 (13) | 17.41* ± 0.40 (13) |

Data are shown as means ± S.E.M. In parenthesis is the number of groups. Each group had 5–7 rats.
*Statistically significant difference ($p < 0.05$) from control group.

Table 2. Percentage increase in body weight of rats fed the three diets during the first 6 weeks

| Diet | Week | | | | | |
|---|---|---|---|---|---|---|
| | 1 | 2 | 3 | 4 | 5 | 6 |
| Control | 32.32 ± 2.08 (45) | 65.20 ± 4.02 (35) | 128.12 ± 5.20 (45) | 171.46 ± 6.38 (45) | 217.40 ± 7.82 (45) | 249.79 ± 8.15 (45) |
| Soybean | 36.75 ± 2.55 (38) | 70.30 ± 5.43 (24) | 141.41 ± 5.58 (38) | 197.58* ± 6.94 (38) | 249.61* ± 9.34 (38) | 286.09* ± 9.94 (38) |
| Fish | 47.57* ± 2.39 (41) | 106.83* ± 2.69 (41) | 162.13* ± 3.32 (41) | 212.88* ± 4.29 (41) | 253.15* ± 5.12 (41) | 285.69* ± 6.06 (41) |

Data are shown as means ± S.E.M. The number of rats is given in parenthesis.
*Statistically significant difference ($p < 0.05$) from control group.

(Table 4). The corticosterone plasma concentration was expressed as μg of corticosterone/dl of plasma. The corticosterone levels of soybean and fish groups, before carrageenin injection, were significantly increased in relation to the control group (Table 4).

*Carrageenin oedema in adrenalectomized groups:* The adrenalectomized control group had a significantly increased oedematous response when compared with control rats. In adrenalectomized soybean and fish groups the observed oedema was significantly increased in relation to intact fish and soybean groups, but these responses were not different from that of adrenalectomized control rats (Fig. 2 and Table 3).

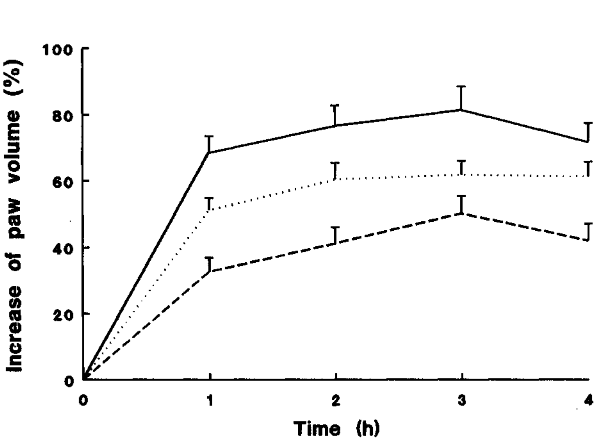

FIG. 1. Carrageenin-induced paw oedema formation in rats fed with control (——), soybean (- -) or fish (····) oil-rich diets.

### Discussion

The alterations in the production of inflammatory mediators and modulators evoked by changes in dietary fatty acid composition might result from changes in membrane fluidity or changes in the release of membrane derived intracellular messengers.[24] In addition to the fact that fatty acids are of great importance in maintaining cell membrane structure, they are key determinants of the behaviour of membrane bound enzymes and receptors.[25]

In the present experiments, fat-fed rats had a higher increase in body weight with a lower food intake than animals fed control chow. In opposition to these results, some authors[26,27] have

Table 3. Oedema formation (% increase of paw volume) in rat paws 1–4 h after carrageenin, in normal and adrenalectomized rats fed the three diets

| Diet | Normal rats | | | | Adrenalectomized rats | | | |
|---|---|---|---|---|---|---|---|---|
| | 1h | 2h | 3h | 4h | 1h | 2h | 3h | 4h |
| Control | 68.65 ± 4.95 (15) | 76.89 ± 6.04 (15) | 81.69 ± 7.00 (15) | 72.11 ± 5.61 (15) | 88.46 ± 6.15 (12) | 119.33 ± 7.08 (12) | 123.86 ± 6.09 (12) | 113.72 ± 5.84 (12) |
| Soybean | 32.86* ± 4.16 (11) | 41.46* ± 4.66 (11) | 50.42* ± 5.20 (11) | 42.30* ± 4.98 (11) | 96.39 ± 7.31 (11) | 114.56 ± 7.80 (11) | 111.62 ± 5.67 (11) | 99.35 ± 5.23 (11) |
| Fish | 51.28*† ± 3.67 (11) | 60.65*† ± 4.89 (11) | 62.13* ± 4.06 (11) | 61.62† ± 4.41 (10) | 71.38 ± 7.57 (10) | 113.00 ± 7.03 (10) | 127.56 ± 7.83 (10) | 115.80 ± 8.78 (10) |

Data are shown as means ± S.E.M. The number of rats is given in parenthesis.
*Statistically different ($p < 0.05$) from control group.
†Statistically different ($p < 0.05$) from soybean group.

Table 4. Corticosterone (μg/dl) and $\alpha_2$-macroglobulin (a.u.) plasma levels in rats, after 6 weeks of diets

| | Control | Soybean | Fish |
|---|---|---|---|
| Corticosterone | 18.89 ± 1.19 (17) | 25.70* ± 1.07 (12) | 27.52* ± 1.42 (14) |
| $\alpha_2$-Macroglobulin | nd (10) | nd (10) | nd (15) |

Data are shown as means ± S.E.M. The number of rats is given in parenthesis. For corticosterone the numbers indicate pools from 2–3 rats. nd = not detectable.
*Statistically significant difference ($p < 0.05$) from control group.

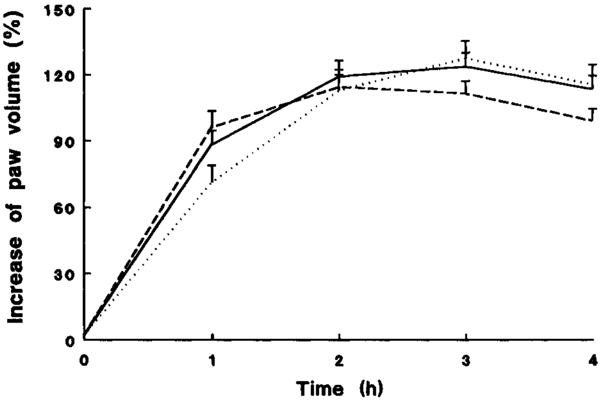

FIG. 2. Carrageenin-induced paw oedema in adrenalectomized rats fed with control (——), soybean (– –) or fish (····) oil-rich diets.

observed a similar evolution of body weight and food intake between rats fed control chow and an n-6 fatty acid-rich diet. These authors, however, studied a lower number of animals and used Purina chow, to which 12% of casein was added as control chow.

In this study, the intake of an n-3 or n-6 PFA rich-diet for approx. 7 weeks caused a significant anti-inflammatory effect on carrageenin oedema in rats. The inflammatory response of the soybean group was even smaller than that of the fish group. Corticosterone levels were higher in fat-fed rats than in control rats, before carrageenin injection. Additionally, adrenalectomy abolished the anti-inflammatory effect observed in the fat-fed animals.

Some studies have associated anti-inflammatory effects with diets high in n-3 PFA[6,7,8] and a reduced production of arachidonic acid metabolites was thought to be responsible for some of these effects. Studying an acute inflammation model, the carrageenin oedema, we could demonstrate not only an anti-inflammatory effect of an n-3 PFA-rich diet but also a strong anti-inflammatory effect of an n-6 PFA-rich diet.

It is difficult to attribute our results to changes in Pg production, since it has been demonstrated that different kinds of Pgs are produced in response to these different fatty acid-rich diets.[2] Moreover, it has been shown that Kupffer cells from rats fed an n-3 PFA rich-diet, when stimulated with lipopolysaccharide (LPS), have a lower production of the pro-inflammatory arachidonic acid metabolites $PgE_2$, $PgI_2$, and $TxA_2$ than Kupffer cells from rats fed an n-6 PFA rich-diet.[28]

We suggested previously[20] that $\alpha_2$-M levels were an important factor accounting for the anti-inflammatory effect observed in the counter-irritation phenomenon, when a second carrageenin oedema was induced 24 h after the first one. Since $\alpha_2$-M is a specific proteinase inhibitor, this putative anti-inflammatory factor[20,29] could contribute to the anti-inflammatory effect observed in fat-fed animals. However, $\alpha_2$-M was not detectable before carrageenin injection in soybean, fish or control rats. This finding demonstrates that the two kinds of fat diets used did not stimulate $\alpha_2$-M synthesis and suggests that this protein does not participate in the anti-inflammatory effect observed during acute inflammation in rats fed fat diets. On the other hand, the elevated corticosterone levels of fat-fed animals, before carrageenin oedema, suggest that this hormone could be one of the factors determining the anti-inflammatory effect observed. To our knowledge, this is the first demonstration of a putative association between corticosterone levels and/or the presence of the adrenal gland and the inflammatory response of rats fed fat-rich diets.

Billiar *et al.*[28] showed a reduction in TNF and IL-1 release by Kupffer cells from rats fed n-3 or n-6 PFA rich-diets. They could not explain these findings by changes in Pg production, since the reduced $PgE_2$ release induced by a fish oil diet would be expected to result in a greater release of cytokines, as a result of a loss of the negative feedback of $PgE_2$.[30] Although these authors[28] have not studied a pure chow-fed control group (not receiving an excess of fat) it is possible that the decrease of these important monokines involved in the inflammatory process may have contributed to the anti-inflammatory effect observed in carrageenin oedema in the present experiments. Some studies have demonstrated that glucocorticoids may not only modulate the function of Kupffer cells and other macrophages but also potentially interact with TNF-α produced by Kupffer cells.[31] Indeed, Kutteh *et al.*[15] showed that LPS-induced production of TNF-α by Kupffer cells can be suppressed by glucocorticoids. In view of these observations it can be speculated that the high corticosteroid levels in our fat-fed animals had an inhibitory effect on the release of important inflammatory mediators, resulting in an anti-inflammatory action of fat diets. Indeed, we found higher corticosteroids levels associated with decreased oedematous response in the fat-fed animals. Moreover, adrenalectomy abolished the inhibitory effect in these groups.

In the present study adrenalectomy increased the oedematous response of all groups. Interestingly, it suppressed the differences in oedema response among controls, soybean and fish groups. These results seem to be correlated with those reported by Baybut and Holsboer.[32] These authors demonstrated, in cultured cells, an inhibitory effect of cortisol on LPS-induced IL-1 release. The removal of cortisol resulted in an increased ability of those cells to synthesize IL-1, which exceeded the response of the control cells that had not been pre-treated with cortisol. Thus, it is possible that the pre-adrenalectomy high levels of corticosteroids may have increased the ability of the adrenalectomized fat-fed rats to produce inflammatory mediators.

The anti-inflammatory response of the soybean group was higher than that of the fish group but the corticosterone levels were not different between them. The absence of a clear relation between corticosterone levels and oedema inhibition indicates that other anti-inflammatory factors, in addition to corticosteroids, may be involved. Additionally, it is possible that soybean-rich diet has a more pronounced effect on inflammatory mediators than that of a fish oil-rich diet in the acute inflammation. Since the anti-inflammatory effect was abolished by adrenalectomy, it is reasonable to think that those putative factors are corticosterone dependent and the adrenal glands are likely to play a role in the phenomenon. Further studies, however, are necessary to determine the precise sequence of events that take place in this complex process.

### References

1. Pike CM. Anti-inflammatory effects of dietary lipid modification. *J Rheumatol* 1989; **16:** 718–720.
2. Zurier B. Essential fatty acids and inflammation. *Ann Rheum Dis* 1991; **50:** 745–746.
3. Lee TH, Mencia-Huerta JM, Shih C, Corey EJ, Lewis RA, Austen KF. Characterization and biologic properties of 5,12-dihydroxy derivatives of eicosapentaenoic acid, including leukotriene $B_5$ and the double lipoxygenase product. *J Biol Chem* 1984; **259:** 2383–2390.
4. Lee TH, Hoover RL, Williams JD, *et al.* Effects of dietary enrichment with eicosapentaenoic and docosahexaenoic acids *in vitro* neutrophil and monocyte leukotriene generation and neutrophil function. *N Engl J Med* 1985; **312:** 1217–1224.
5. Darlington LG. Do diets rich in polyunsaturated fatty acids affect disease activity in rheumatoid arthritis? *Ann Rheum Dis* 1988; **47:** 169–172.
6. Kremer JM, Biganoette J, Michalek AV, *et al.* The effects of manipulation of dietary fatty acids on clinical manifestations of rheumatoid arthritis. *Lancet* 1985; **1:** 184–187.
7. Leslie CA, Gonnerman WA, Ullman MD, Hayes KC, Franzblace C, Cathcart ES. Dietary fish oil modulates fatty acids and decreases arthritis susceptibility in mice. *J Exp Med* 1985; **162:** 1336–1349.
8. Cathcart ES, Mortensen RF, Leslie CA, Conte JM, Gonnerman WA. A fish oil diet inhibits amyloid P component (AP) acute phase responses in arthritis in susceptible mice. *J Immunol* 1987; **139:** 89–91.
9. Kromhout D, Bosschieter EB, Coulander C de L. The inverse relation between fish consumption and 20-year mortality from coronary heart disease. *N Engl J Med* 1985; **312:** 1205–1209.
10. Prickett JD, Trentham DE, Robinson DR. Dietary fish oil augments the induction of arthritis in rats immunized with type II collagen. *J Immunol* 1984; **132:** 725–729.
11. Endres S, Ghorbani R, Kelley VE, *et al.* The effect of dietary supplementation with n-3 polyunsaturated fatty acids on the synthesis of interleukin-1 and tumor necrosis factor by mononuclear cells. *N Engl J Med* 1989; **320:** 265–271.
12. Andreis PG, Neri G, Belloni AS, Mazzocchi G, Kasprzak A, Nussdorfer GG. Interleukin-1β enhances corticosterone secretion by acting directly on the rat adrenal gland. *Endocrinology* 1991; **129:** 53–57.
13. Gwosdow AR, O'Connell NA, Spencer JA, *et al.* Interleukin-1 induced corticosterone release occurs by an adrenergic mechanism from rat adrenal gland. *Am J Physiol* 1992; **163:** E461–E466.
14. Baybutt HN, Holsboer F. Inhibition of macrophage differentiation and function by cortisol. *Endocrinology* 1990; **127;** 476–480.
15. Kutteh Wh, Rainey WE, Carr BR. Glucocorticoids inhibit lipopolysaccharide induced production of tumor necrosis factor-α by human fetal Kupffer cells. *J Clin Endocrinol Metab* 1991; **73:** 296–301.
16. Fleetwood MK, Gander GW, Goodale F. Effect of metabolic inhibitors on pyrogen production by rabbit leukocytes. *Proc Soc Exp Biol* 1975; **149:** 336–339.
17. Errasfa M, Rothhut B, Fradin A, *et al.* The presence of lipocortin in human skin fibroblasts and regulation by anti-inflammatory steroids. *Biochim Biophys Acta* 1985; **874:** 247–254.
18. Davidson FF, Dennis EA, Powell M, Glenney JR. Inhibition of phospholipase $A_2$ by 'lipocortins' and calpactins: an effect of binding to substrate phospholipids. *J Biol Chem* 1987; **262:** 1698–1705.
19. Silveira VLF, Limãos EA. Effect of bacterial endotoxin on plasma concentration of haptoglobin and fibrinogen in rats treated with metopyrone. *Agents and Actions* 1990; **31:** 143–147.
20. Silveira VLF, Limãos EA. Participation of $\alpha_2$-macroglobulin in counter-irritation. *Agents and Actions* 1992; **36:** 294–298.
21. Guillemin R, Clayton GW, Lipscomb HS, Smith JD. Fluorometric measurement of rat plasma and adrenal corticosterone concentration. *J Lab Clin Med* 1959; **53:** 830–832.
22. Mancini G, Carbonara AD, Heremans JF. Immunochemical quantitation of antigens by single radial immunodiffusion. *Immunochemistry* 1965; **2:** 235–254.
23. Zarrow MX, Yochim JM, McCarthy JL. The adrenal corticoids. In: Zarrow MX, ed. *A Sourcebook of Basic Techniques.* Academic Press: New York, London, 1964; 177–223.
24. Stubbs CD, Smith AD. The modification of membrane polyunsaturated fatty acid composition in relation to membrane fluidity and function. *Biochem Biophys Acta* 1984; **779:** 89–137.
25. McMurchie EJ. Dietary lipids and the regulation of membrane fluidity and function. In: Aloid RC, Curtain CC, Gordon LM, eds. *Physiological regulation of membrane fluidity.* New York: Alan R Liss, 1988; 189–237.
26. Guimarães ARP, Sitnik RH, Nascimento Curi CMPO, Curi R. Polyunsaturated and saturated fatty acids-rich diets and immune tissue. 2. Maximal activities of key enzymes of glutaminolysis, glycolysis, pentose-phosphate-pathway and Krebs cycle in thymus, spleen and mesenteric lymph nodes. *Biochem Int* 1990; **22:** 1015–1023.
27. Egami MI, Guimarães APR, Nascimento Curi CMPO, Curi R. Effect of fatty acid-rich diets on thymocyte proliferation and thymus involution during growing. *Physiol Behav* 1993; **53:** 531–534.
28. Billiar TR, Bankey PE, Syingen BA, *et al.* Fatty acid intake and Kupffer cell function: fish oil alters eicosanoid and monokine production to endotoxin stimulation. *Surgery* 1988; **104:** 343–349.
29. Van Gool J, Shreuder J, Ladiges NCJJ. Inhibitory effect of fetal $\alpha_2$-globulin, an acute phase protein on carrageenin oedema in the rat. *J Path* 1974; **112:** 245–262.
30. Kunkel SL, Chensue SW, Phan SH. Prostaglandins as endogenous mediators of interleukin 1 production. *J Immunol* 1986; **136:** 186–192.
31. Silen ML, Hesse DG, Felsen D, *et al.* Cachectin/tumor necrosis factor production by fetal and newborn rat hepatic macrophages. *J Pediatr Surg* 1989; **24:** 34–38.
32. Baybutt HN, Holsboer F. Inhibition of macrophage differentiation and function by cortisol. *Endocrinology* 1990; **127:** 476–480.

ACKNOWLEDGEMENTS. The authors are grateful to Dr E. B. Ribeiro for help in the preparation of the manuscript and for helpful discussions. V. L. F. Silveira and E. A. Limãos are recipients of fellowships from CNPq and D. W. Nunes is a recipient of a PIBIC fellowship.

**Received 19 June 1995; accepted 22 June 1995**
